# Supplementary material for: Flux-dependent graphs for metabolic networks
Source: NPJ Syst Biol Appl. 2018 Aug 14;4:32. doi: 10.1038/s41540-018-0067-y (PMC6092364; doi:10.1038/s41540-018-0067-y)
Supplement: Supplementary file 1 — Supplementary Material [file 41540_2018_67_MOESM1_ESM.pdf]

Flux-dependent graphs for metabolic networks

Mariano Beguerisse-Díaz,[1,2,*] Gabriel Bosque,[3,†] Diego Oyarzún,[2] Jesús Picó,[3] and Mauricio Barahona[2,‡]

[1]*Mathematical Institute, University of Oxford, Oxford OX2 6GG, UK*

[2]*Department of Mathematics, Imperial College London, London, SW7 2AZ, UK*

[3]*Institut Universitari d'Automàtica i Informàtica Industrial, Universitat Politècnica de València, Camí de Vera s/n, 46022 Valencia, Spain.*

Supplementary Information

Appendix SI 1: Relation of the NFG with a directed version of the RAG

A directed version of the RAG 3 could in principle be obtained from the boolean production/consumption matrices $\widehat{\mathbf{S}}_{2m}^{+}$ and $\widehat{\mathbf{S}}_{2m}^{-}$ as follows. Projecting onto the space of reactions gives the $2m \times 2m$ (asymmetric) adjacency matrix

$$\mathbf{D} = \widehat{\mathbf{S}}_{2m}^{+\,T}\,\widehat{\mathbf{S}}_{2m}^{-}, \tag{SI 1}$$

where the entries D_{ij} represent the total number of metabolites *produced* by reaction R_i that are *consumed* by reaction R_j. A directed version of the Reaction Adjacency Graph on m nodes (directly comparable to the standard RAG) is then

$$\mathbf{A}_{\text{dir}} = \begin{bmatrix}\mathbf{I}_m & \mathbf{I}_m\end{bmatrix}\mathbf{D}\begin{bmatrix}\mathbf{I}_m \\ \mathbf{I}_m\end{bmatrix}. \tag{SI 2}$$

Clearly, when the metabolic model contains only reversible reactions, (i.e., the reversibility vector is all ones, $\mathbf{r} = \mathbb{1}_m$), it follows that $\mathbf{A}_{\text{dir}} = \mathbf{A}$.

Although $\mathbf{A}_{\text{dir}}$ does not include spurious edges introduced by non-existent backward reactions, its structure is still obscured by the effect of uninformative connections created by pool metabolites.

Appendix SI 2: Details of the toy metabolic network

As an illustration of the graph construction, the toy metabolic network in Fig. 1 was taken from Ref. [1]. The graph matrices for this model are as follows:

- Reaction Adjacency Graph, Eq. 3:

$$\mathbf{A} = \widehat{\mathbf{S}}^T\widehat{\mathbf{S}} = \begin{bmatrix} 1&1&0&0&0&0&0&0\\ 1&2&1&1&0&0&0&0\\ 0&1&2&1&1&0&0&1\\ 0&1&1&2&0&1&1&1\\ 0&0&1&0&2&1&0&2\\ 0&0&0&1&1&2&1&2\\ 0&0&0&1&0&1&1&1\\ 0&0&1&1&2&2&1&3 \end{bmatrix}.$$

* beguerisse@maths.ox.ac.uk

† gabbosch@upv.es

‡ m.barahona@imperial.ac.uk

- Normalised Flow Graph, Eq. 8:

$$\boldsymbol{\mathcal{D}} = \frac{1}{n}\mathbf{S}_{2m}^{+\,T}\left(\mathbf{W}_{+}^{\dagger}\mathbf{W}_{-}^{\dagger}\right)\mathbf{S}_{2m}^{-} = \left[\begin{array}{c|ccccccccc} & R_1 & R_2 & R_3 & R_4 & R_5 & R_6 & R_7 & R_8 & R_{4r} \\ R_1 & 0 & 0.2 & 0 & 0 & 0 & 0 & 0 & 0 & 0 \\ R_2 & 0 & 0 & 0.05 & 0.05 & 0 & 0 & 0 & 0 & 0 \\ R_3 & 0 & 0 & 0 & 0 & 0.1 & 0 & 0 & 0.1 & 0 \\ R_4 & 0 & 0 & 0 & 0 & 0 & 0.04 & 0.04 & 0.08 & 0.04 \\ R_5 & 0 & 0 & 0 & 0 & 0 & 0 & 0 & 0.1 & 0 \\ R_6 & 0 & 0 & 0 & 0 & 0 & 0 & 0 & 0.1 & 0 \\ R_7 & 0 & 0 & 0 & 0 & 0 & 0 & 0 & 0 & 0 \\ R_8 & 0 & 0 & 0 & 0 & 0 & 0 & 0 & 0 & 0 \\ R_{4r} & 0 & 0 & 0.05 & 0.05 & 0 & 0 & 0 & 0 & 0 \end{array}\right].$$

- Mass Flow Graph for FBA scenario 1, Eq. 12:

$$\begin{array}{rrrr} & \mathbf{v}_{\mathrm{lb}_1} & \mathbf{v}_{\mathrm{ub}_1} & \mathbf{v}_1^* \\ R_1: & 10 & 10 & 10 \\ R_2: & 0 & 10 & 10 \\ R_3: & 0 & 10 & 4.992 \\ R_4: & -10 & 10 & 5.008 \\ R_5: & 0 & 10 & 2.492 \\ R_6: & 0 & 10 & 0.008 \\ R_7: & 0 & 10 & 0 \\ R_8: & 0 & 10 & 2.5 \\ R_{4r}: & -10 & 10 & 0 \end{array} \qquad \mathbf{M}(\mathbf{v}_1^*) = \left[\begin{array}{c|ccccccc} & R_1 & R_2 & R_3 & R_4 & R_5 & R_6 & R_8 \\ R_1 & 0 & 10 & 0 & 0 & 0 & 0 & 0 \\ R_2 & 0 & 0 & 4.992 & 5.008 & 0 & 0 & 0 \\ R_3 & 0 & 0 & 0 & 0 & 2.492 & 0 & 2.5 \\ R_4 & 0 & 0 & 0 & 0 & 0 & 0.008 & 5 \\ R_5 & 0 & 0 & 0 & 0 & 0 & 0 & 2.492 \\ R_6 & 0 & 0 & 0 & 0 & 0 & 0 & 0.008 \\ R_8 & 0 & 0 & 0 & 0 & 0 & 0 & 0 \end{array}\right].$$

- Mass Flow Graph for FBA scenario 2, , Eq. 12:

$$\begin{array}{rrrr} & \mathbf{v}_{\mathrm{lb}_2} & \mathbf{v}_{\mathrm{ub}_2} & \mathbf{v}_2^* \\ R_1: & 10 & 10 & 10 \\ R_2: & 0 & 10 & 10 \\ R_3: & 0 & 10 & 3.877 \\ R_4: & -10 & 10 & 6.123 \\ R_5: & 0 & 10 & 1.877 \\ R_6: & 0 & 10 & 0.123 \\ R_7: & 2 & 10 & 2 \\ R_8: & 0 & 10 & 2 \\ R_{4r}: & -10 & 10 & 0 \end{array} \qquad \mathbf{M}(\mathbf{v}_2^*) = \left[\begin{array}{c|cccccccc} & R_1 & R_2 & R_3 & R_4 & R_5 & R_6 & R_7 & R_8 \\ R_1 & 0 & 10 & 0 & 0 & 0 & 0 & 0 & 0 \\ R_2 & 0 & 0 & 3.877 & 6.123 & 0 & 0 & 0 & 0 \\ R_3 & 0 & 0 & 0 & 0 & 1.877 & 0 & 0 & 2 \\ R_4 & 0 & 0 & 0 & 0 & 0 & 0.123 & 2 & 4 \\ R_5 & 0 & 0 & 0 & 0 & 0 & 0 & 0 & 1.877 \\ R_6 & 0 & 0 & 0 & 0 & 0 & 0 & 0 & 0.123 \\ R_7 & 0 & 0 & 0 & 0 & 0 & 0 & 0 & 0 \\ R_8 & 0 & 0 & 0 & 0 & 0 & 0 & 0 & 0 \end{array}\right].$$

Appendix SI 3: Reaction communities in context-free graphs of the core *E. coli* metabolic model

1. Reaction Adjacency Graph, A

A robust partition into seven communities in the RAG was found at Markov time $t = 6.01$ (Fig. SI 1A). The communities at this resolution (Fig. 3B) are:

- Community C1(**A**) contains all the reactions that consume or produce ATP and water (two pool metabolites). Production of ATP comes mostly from oxidative phosphorylation (ATPS4r) and substrate level phosphorylation reactions such as phosphofructokinase (PFK), phosphoglicerate kinase (PGK) and succinil-CoA synthase (SUCOAS). Reactions that consume ATP include glutamine synthetase (GLNS) and ATP maintenance equivalent reaction (ATPM). The reactions L-glutamine transport via ABC system (GLNabc), acetate transport in the form of phosphotransacetilase (PTAr), and acetate kinase (ACKr) are also part of this community. Additionally, C1(**A**) (green) contains also reactions that involve H_2O. Under normal conditions water is assumed to be abundant in the cell, thus the biological link that groups these reactions together is tenuous.
- Community C2(**A**) includes the reactions NADH dehydrogenase (NADH16), cytochrome oxidase (CYTBD), and transport and exchange reactions. These two reactions involve pool metabolites (such as H^+) which create a large number of connection. Other members include fumarate reductase (FR7) and succinate dehydrogenase (SUCDi) which couple the TCA cycle with the electron transport chain (through ubiquinone-8 reduction and ubiquinol-8 oxidation). Reactions that include export and transport of most secondary carbon sources (such as

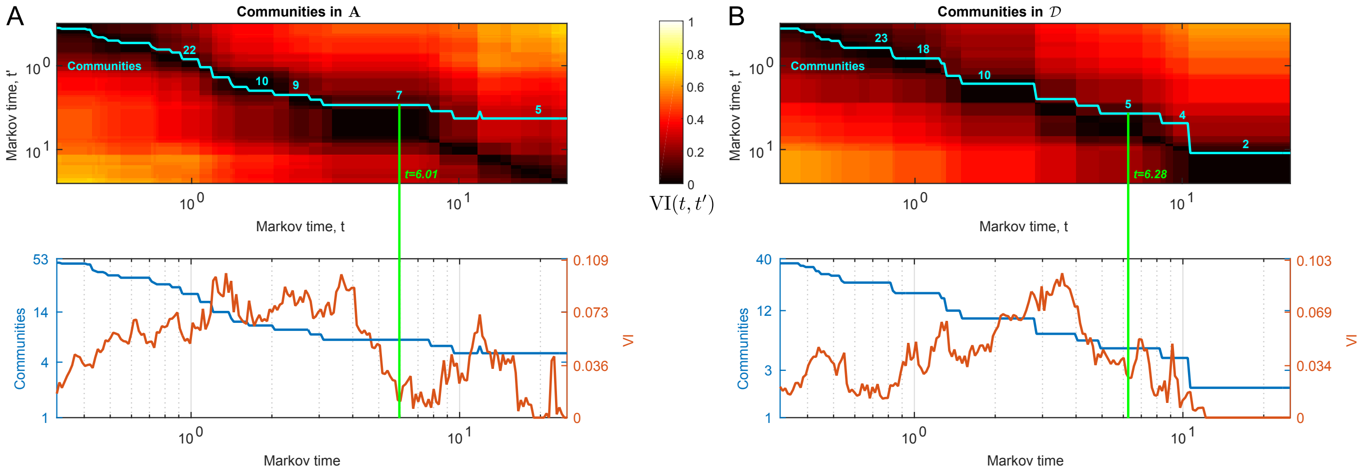

FIG. SI 1. Community structure in the template networks A and 𝓓. (A) Communities in **A**. Top plot: Variation of Information (VI) of the best partition found at Markov time t with every other partition at time t'. Bottom plot: Number of communities and VI of the ensemble of solutions found at each Markov time. A robust partition into seven communities is found at $t = 6.01$. (B) Communities and VI in **𝓓**. A robust partition into five communities is found at $t = 6.28$.

pyruvate, ethanol, lactate, acetate, malate, fumarate, succinate or glutamate) are included in the community as well. These reactions are included in the community because of their influence in the proton balance of the cell. Most of these reactions do not occur under normal circumstances. This community highlights the fact that in the absence of biological context, many reactions that do not normally interact can be grouped together.

- Community C3(**A**) contains reactions that produce or consume nicotinamide adenine dinucleotide (NAD^+), nicotinamide adenine dinucleotide phosphate ($NADP^+$), or their reduced variants NADH and NADPH. The main two reactions of the community are NAD(P) transhydrogenase (THD2) and NAD^+ transhydrogenase (NADTRHD). There are also reactions related to the production of NADH or NADPH in the TCA cycle such as isocitrate dehydrogenase (ICDHyr), 2-oxoglutarate dehydrogenase (AKGDH) and malate dehydrogenase (MDH). The community also includes reactions that are not frequently active such as malic enzime NAD (ME1) and malic enzime NADH (ME2) or acetate dehydrogenase (ACALD) and ethanol dehydrogenase (ALCD2x).

- Community C4(**A**) contains the main carbon intake of the cell (glucose), the initial steps of glycolysis, and most of the pentose phosphate shunt. These reactions are found in this community because the metabolites involved in these reactions (e.g., alpha-D-ribose-5-phosphate (r5p) or D-erythrose-4-phosphate (e4p)) are only found in these reactions. This community includes the biomass reaction due to the number of connections created by growth precursors.

- Communities C5(**A**), C6(**A**) and C7(**A**) are small communities that contain oxygen intake, ammonium intake and acetaldehyde secretion reactions, respectively.

2. Normalised Flow Graph, 𝓓

A robust partition into five communities in the NFG was found at Markov time $t = 6.28$ (Fig. SI 1B). The communities at this resolution (Fig. 3C) are:

- Community C1(**𝓓**) includes the first half of the glycolysis and the complete pentose phosphate pathway. The metabolites that create the connections among these reactions such as D-fructose, D-glucose, or D-ribulose.

- Community C2(**𝓓**) contains the main reaction that produces ATP through substrate level (PGK, PYK, ACKr) and oxidative phosphorylation (ATPS4r). The flow of metabolites among the reactions in this community includes some pool metabolites such as ATP, ADP, H_20, and phosphate. However, there are connections created by metabolites that only appear in a handful of reactions such as adenosine monophosphate (AMP) whose

sole producer is phosphoenolpyruvate synthase (PPS) and its sole consumer is ATPS4r. This community also contains the biomass reaction.

- Community C3($\boldsymbol{\mathcal{D}}$) includes the core of the citric acid (TCA) cycle such as citrate synthase (CS), aconitase A/B (ACONTa/b), and anaplerotic reactions such as malate synthase (MALS), malic enzyme NAD (ME1), and malic enzyme NADP (ME2). This community also includes the intake of cofactors such as CO_2.
- Community C4($\boldsymbol{\mathcal{D}}$) contains reactions that are secondary sources of carbon such as malate and succinate, as well as oxidative phosphorilation reactions.
- Community C5($\boldsymbol{\mathcal{D}}$) contains some reactions part of the pyruvate metabolism subsystem such as D-lactate dehydrogenase (LDH-D), pyruvate formate lyase (PFL) or acetaldehyde dehydrogenase (ACALD). In addition, it also includes the tranport reaction for the most common secondary carbon metabolites such as lactate, formate, acetaldehyde and ethanol.

Appendix SI 4: Reaction communities in Mass Flow Graphs of *E. coli* metabolism under different biological scenarios

1. $\mathbf{M}_{\mathrm{glc}}$: aerobic growth under glucose

This graph has 48 reactions with nonzero flux and 227 edges. At Markov time $t = 7.66$ (Fig. SI 2A) this graph has a partition into three communities (Fig. 4A):

- Community C1($\mathbf{M}_{\mathrm{glc}}$) comprises the intake of glucose and most of the glycolysis and pentose phosphate pathway. The function of the reactions in this community consists of carbon intake and processing glucose into phosphoenolpyruvate (PEP). This community produces essential biocomponents for the cell such as alpha-D-Ribose 5-phosphate (rp5), D-Erythrose 4-phosphate (e4p), D-fructose-6-phosphate (f6p), glyceraldehyde-3-phosphate (g3p) or 3-phospho-D-glycerate (3pg). Other reactions produce energy ATP and have reductive capabilities for catabolism.
- Community C2($\mathbf{M}_{\mathrm{glc}}$) contains the electron transport chain which produces the majority of the energy of the cell. In the core *E coli* metabolic model the chain is represented by the reactions NADH dehydrogenase (NADH16), cytochrome oxidase BD (CYTBD) and ATP synthase (ATPS4r). This community also contains associated reactions to the electron transport such as phosphate intake (EXpi(e), PIt2), oxygen intake (EXo2(e), O2t) and proton balance (EXh(e)). This community also includes the two reactions that represent energy maintenance costs (ATPM), and growth (biomass); this is consistent with the biological scenario because ATP is the main substrate for both ATPM, and the biomass reaction.
- Community C3($\mathbf{M}_{\mathrm{glc}}$) contains the TCA cycle at its core. The reactions in this community convert PEP into ATP, NADH and NADPH. In contrast with C1($\mathbf{M}_{\mathrm{glc}}$), there is no precursor formation here. Beyond the TCA cycle, pyruvate kinase (PYK), phosphoenolpyruvate carboxylase (PPC) and pyruvate dehydrogenase (PDH) appear in this community. These reactions highlight the two main carbon intake routes in the cycle: oxalacetate from PEP through phosphoenol pyruvate carboxylase (PPC), and citrate from acetyl coenzyme A (acetyl-CoA) via citrate synthase (CS). Furthermore, both routes begin with PEP, so it is natural for them to belong to the same community along with the rest of the TCA cycle. Likewise, the production of L-glutamate from 2-oxoglutarate (AKG) by glutamate dehydrogenase (GLUDy) is strongly coupled to the TCA cycle.

2. $\mathbf{M}_{\mathrm{etoh}}$: aerobic growth under ethanol

This graph contains 49 reactions and 226 edges. At Markov time $t = 6.28$ (Fig. SI 2B) this graph has a partition into three communities (Fig. 4B):

- Community C1($\mathbf{M}_{\mathrm{etoh}}$) in this graph is similar to its counterpart in $\mathbf{M}_{\mathrm{glc}}$, but with important differences. For example, the reactions in charge of the glucose intake (EXglc(e) and GLCpts) are no longer part of the network (i.e., they have zero flux), and reactions such as malic enzyme NAPD (ME2) and phosphoenolpyruvate caboxykinase (PPCK), which now appear in the network, belong to this community. This change in the network reflect the cell's response to a new biological situation. The carbon intake through ethanol has changed the direction of glycolysis into gluconeogenesis [2] (the reactions in C1($\mathbf{M}_{\mathrm{glc}}$) in Fig. 4A are now operating in the reverse direction

in Fig. 4B). The main role of the reactions in this community is the production of bioprecursors such as PEP, pyruvate, 3-phospho-D-glycerate (3PG) glyceraldehyde-3-phosphate (G3P), D-fructose-6-phosphate (F6P), and D-glucose-6-phosphate, all of which are substrates for growth. Reactions ME2 and PPCK also belong to this community due to their production of PYR and PEP. Reactions that were in a different community in $\mathbf{M}_{\mathrm{glc}}$, such as GLUDy and ICDHyr which produce precursors L-glutamate and NADPH respectively, are now part of C1($\mathbf{M}_{\mathrm{etoh}}$). This community also includes the reactions that produce inorganic substrates of growth such as NH_4, CO_2 and H_2O.

- Community C2($\mathbf{M}_{\mathrm{etoh}}$) contains the electron transport chain and the bulk of ATP production, which is similar to C2($\mathbf{M}_{\mathrm{glc}}$). However, there are subtle differences that reflect changes in this new scenario. Ethanol intake and transport reactions (EXetoh(e) and ETOHt2r) appear in this community due to their influence in the proton balance of the cell. In addition, C2($\mathbf{M}_{\mathrm{etoh}}$) contains NADP transhydrogenase (THD2) which is in charge of NADH/NADPH balance. This reaction is present here due to the NAD consumption involved in the reactions ACALD and ethanol dehydrogenase (ALCD2x), which belong to this community as well.

- Community C3($\mathbf{M}_{\mathrm{etoh}}$) contains most of the TCA cycle. The main difference between this community and C1($\mathbf{M}_{\mathrm{glc}}$) is that here acetyl-CoA is extracted from acetaldehyde (which comes from ethanol) by the reaction acetaldehyde dehydrogenase reaction (ACALD), instead of the classical pyruvate from glycolysis. The glycoxylate cycle reactions isocitrate lyase (ICL) and malate synthase (MALS) which now appear in the network, also belong to this community. These reactions are tightly linked to the TCA cycle and appear when the carbon intake is acetate or ethanol to prevent the loss of carbon as CO_2.

3. $\mathbf{M}_{\mathrm{anaero}}$: anaerobic growth

This graph contains 47 reactions and 212 edges. At Markov time $t = 6.01$ (Fig. SI 2C) this graph has a partition into four communities (Fig. 4C):

- Community C1($\mathbf{M}_{\mathrm{anaero}}$) contains the reactions responsible D-glucose intake (EXglc) and most of the glycolysis. The reaction that represents the cellular maintenance energy cost, ATP maintenance requirement (ATPM), is included in this community because of the increased strength of its connection to the substrate-level phosphorilation reaction phosphoglycerate kinase (PGK). Also note that reactions in the pentose phosphate pathway do not belong to the same community as the glycolysis reactions (unlike in $\mathbf{M}_{\mathrm{glc}}$ and $\mathbf{M}_{\mathrm{etoh}}$).

- Community C2($\mathbf{M}_{\mathrm{anaero}}$) contains the conversion of PEP into formate through the sequence of reactions PYK, PFL, FORti and EXfor(e). More than half of the carbon secreted by the cell becomes formate.

- Community C3($\mathbf{M}_{\mathrm{anaero}}$) includes the biomass reaction and the reactions in charge of supplying it with substrates. These reactions include the pentose phosphate pathway (now detached from C1($\mathbf{M}_{\mathrm{glc}}$)), which produce essential growth precursors such as alpha-D-ribose-5-phosphate (r5p) or D-erythrose-4-phosphate (e4p). The TCA cycle is present as well because its production of two growth precursors: 2-oxalacetate and NADPH. Finally, the reactions in charge of acetate production (ACKr, ACt2r and EXac(e)) are also members of this community through the ability of ACKr to produce ATP. Glutamate metabolism reaction GLUDy is also included in this community. It is worth mentioning that the reverse of ATP synthase (ATPS4r) is present in this community because here, unlike in $\mathbf{M}_{\mathrm{glc}}$, ATPS4r consumes ATP instead of producing it. When this flux is reversed, then ATPS4r is in part responsible for pH homeostasis.

- Community C4($\mathbf{M}_{\mathrm{anaero}}$) includes the main reactions involved in NADH production and consumption, which occurs via glyceraldehyde-3-phosphate dehydrogenase (GAPD). NADH consumption occurs in two consecutive steps in ethanol production: in ACALD and ALCD2x. The phosphate intake and transport reactions EXpi(e) and PIt2r belong to this community because most of the phosphate consumption takes place at GAPD. Interestingly, the core reaction around which the community forms (GAPD) is not present in the community. It is included in earlier Markov times but when communities start to get larger the role of GAPD becomes more relevant as a part of the glycolysis than its role as a NADH hub. This is a good example of how the graph structure and the clustering method are able to capture two different roles in the same metabolite.

4. $\mathbf{M}_{\mathrm{lim}}$: aerobic growth under limiting conditions

This graph has 52 nodes and 228 edges. At Markov time $t = 13$ this graph (Fig. SI 2D) has a partition into three communities (Fig. 4D):

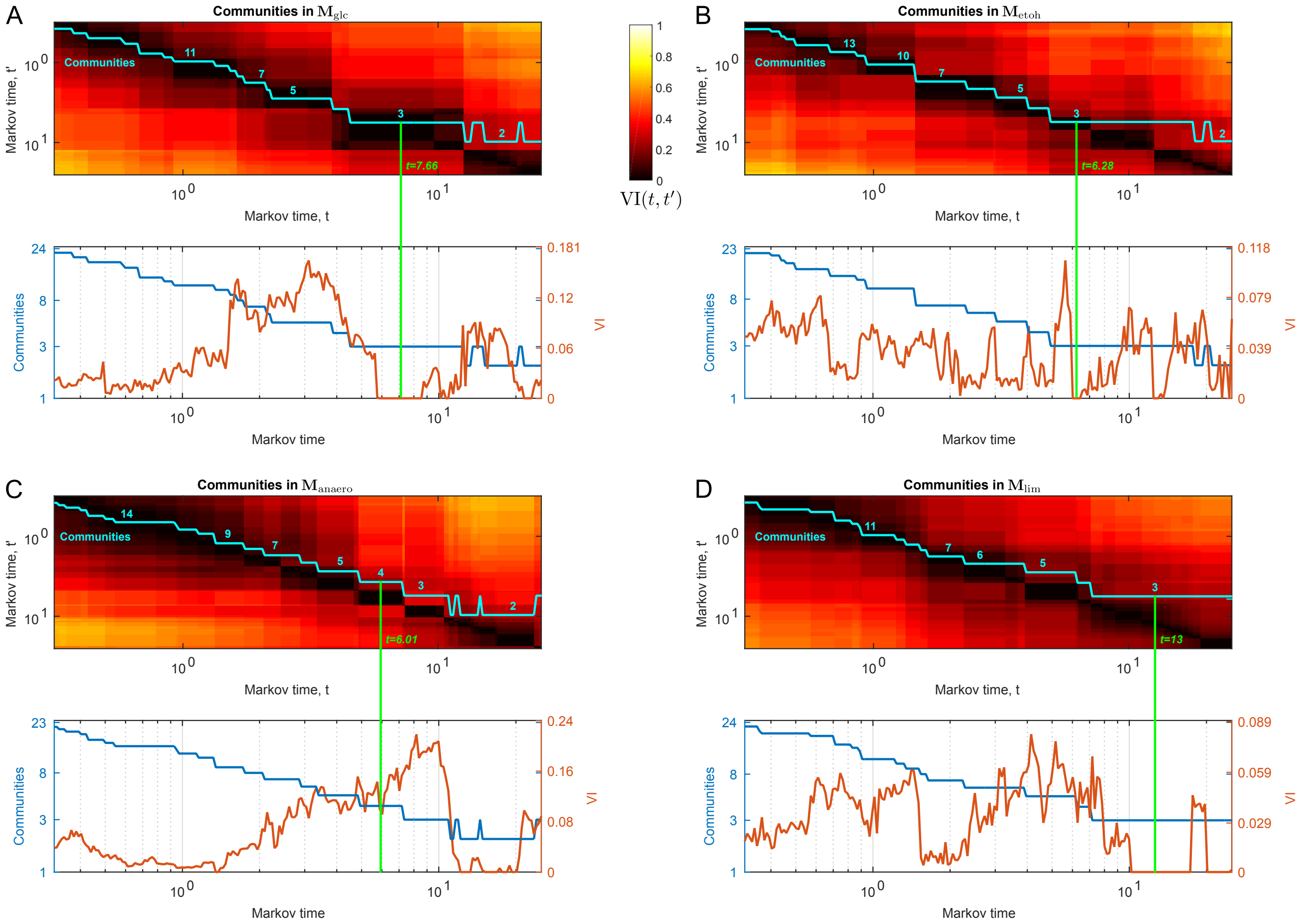

FIG. SI 2. Community structure in the MFGs. Number of communities and VI of the MFGs in four biological scenarios. (A) The graph $\mathbf{M}_{glc}$ has a robust partition into three communities at $t = 7.66$. (B) $\mathbf{M}_{etoh}$ has a partition into three communities at $t = 6.28$. (C) $\mathbf{M}_{anaero}$ has four communities at $t = 6.01$. (D) $\mathbf{M}_{lim}$ has three communities at $t = 13.0$.

- Community C1($\mathbf{M}_{lim}$) contains the glycolysis pathway (detached from the pentose phosphate pathway). This community is involved in precursor formation, ATP production, substrate-level phosphorylation and processing of D-glucose into PEP.

- Community C2($\mathbf{M}_{lim}$) contains the bioenergetic machinery of the cell; the main difference to the previous scenarios is that the electron transport chain has a smaller role in ATP production (ATPS4r), and substrate-level phosphorylation (PGK, PYK, SUCOAS, ACKr) becomes more important. In $\mathbf{M}_{lim}$ the electron transport chain is responsible for the 21.8% of the total ATP produced in the cell while in $\mathbf{M}_{glc}$ it produces 66.5%. The reactions in charge of intake and transport of inorganic ions such as phosphate (EXpi(e) and PIt2r), O_2 (EXO_2(e) and O_2t)and H_2O (EXH_2O and H_2Ot) belong to this community as well. This community includes the reactions in the pentose phosphate pathway that produce precursors for growth: transketolase (TKT2) produces e4p, and ribose-5-phosphate isomerase (RPI) produces r5p.

- Community C3($\mathbf{M}_{lim}$) is the community that differs the most from those in the other aerobic growth networks ($\mathbf{M}_{glc}$ and $\mathbf{M}_{etoh}$). This community gathers reactions that under normal circumstances would not be so strongly related but that the limited availability of ammonium and phosphate have forced together; its members include reactions from the TCA cycle, the pentose phosphate pathway, nitrogen metabolism and by-product secretion. The core feature of the community is carbon secretion as formate and acetate. Reactions PPC, malate dehydrogenase (MDH) reverse and ME2 channel most of the carbon to the secretion routes in the form of formate and acetate. The production of L-glutamine seems to be attached to this subsystem through the production of

NADPH in ME2 and its consumption in the glutamate dehydrogenase NAPD (GLUDy).

---

[1] Rabinowitz, J. D. & Vastag, L. Teaching the design principles of metabolism. *Nat Chem Biol* **8**, 497–501 (2012). URL `http://dx.doi.org/10.1038/nchembio.969`.
[2] Berg, J., Tymoczko, J. & Stryer, L. *Biochemistry, Fifth Edition* (W. H. Freeman, 2002).
